# Cultural and Socio-Economic Factors on Changes in Aging among Iranian Women

**DOI:** 10.5539/gjhs.v6n3p145

**Published:** 2014-02-28

**Authors:** Masoumeh Bagheri-Nesami, Seyed Afshin Shorofi

**Affiliations:** 1 Department of Medical - Surgical Nursing, School of Nursing & Midwifery, Mazandaran University of Medical Sciences, Sari, Iran

**Keywords:** women, community dwelling elderly, cultural context, socioeconomic factors

## Abstract

The aim of the study is to determine the cultural and socio-economic factors that influence changes in aging among Iranian women. This qualitative study was part of a more extensive study designed according to grounded theory method. A purposeful, snowball and theoretical sampling technique was used. Data collection instruments were interviews and field notes. Duration of interviews differed and ranged from 38 to 110 minutes. Data collection process, coding and analysis were performed simultaneously. Collected data were analyzed using the recommended method by Corbin and Straus (1998 and 2008). The factors were formed from 6 subcategories: cultural and socio-economic status in the past, urban/rural life, companionship status, beliefs and attitudes, higher responsibilities of women and women’s financial capability. This study explained the various aspects of cultural and socio-economic changes in the elderly participants based on their real experiences.

## 1. Introduction

It has been stated that by 2030, the elderly will constitute 21% of the total world population (Strydom, 2005). According to biostatistics estimations, by the year 1410 AP (2030 AD), Iran will experience an explosion in aging, placing 20 to 25 percent of the population over the age of sixty (Foroughan, 2001).

The increasing elderly population around the world is one of the most challenging issues in the health and welfare fields and aging is discussed as a universal phenomenon (Gates, 2000). Aging and the aging process are often associated with increased chronic diseases. It also seems that inactivity and general disability are part of the aging process. Most of these problems lead to other issues such as social isolation, functional loss, economic loss and depression (Easley & Schaller, 2003). In addition, older age is associated with higher risk of disease, physical and emotional damage, decrease of functional capacity, multiplicity and abundance of problems related to aging, all of which are natural phenomenon (Strydom, 2005). The results of studies confirm that aging puts older people at risk of further damage. On the other hand, elder women have low income and low socio-economic status that makes them vulnerable to be placed in this category (Cyr, 2007). Clearly the changes in the framework of aging are influenced by internal and external factors (Balcombe & Sinclair 2001). These include a wide range of factors influencing physical, mental, emotional, cultural and social-economic aspects. Several studies have reported the effects of various factors including culture, occupation, education and low income on physical disabilities (Chiu, Hsieh, Mau & Lee, 2005; Day, 2008; Jacelon, Connelly, Brown, Proulx & Vo, 2004), mental health (Grundy & Sloggett, 2003; Kawachi & Berkman, 2001), depression (Black, White & Hannum, 2007), life satisfaction (Nagalingam, 2007), health status, having healthy aging (Bryant, Corbett & Kutner, 2001; Hinck, 2004; Jamjan, Maliwan, Pasunant, Sirapo-Ngam & Porthiban, 2002; Shin, Kang, Park, Cho & Heitkemper, 2008; Strydom, 2005; van Manen, 2006) and successful aging (Matsubayashi, Ishine, Wada & Okumiya, 2006). Moreover, some researchers showed that attitudes, expectations and behavior are formed and influenced by culture and affect aging via adopted behavior (Bryant et al., 2001; Ramamurti, 1997; Reichstadt, Depp, Palinkas, Folsom, & Jeste, 2007; Sarkisian, Hays & Mangione, 2002). For instance, the dominant lifestyle in rural areas is being active and working hard. Also, several studies have demonstrated the effect of rural lifestyle on the health of the elderly (Dorfman, Murty, Evans, Ingram & Power, 2004; Smith, 2007).

As is the case in many developing countries, the senior population in Iran is significantly growing. The baby boom occurred in war-time Iran (1980s), and it is predicted that more than 26 million seniors (over 60 years old) will live in Iran by 2050. The aged people (65 years and older) represent more than 7% of the population, and it is projected to be more than 10% and 21% by 2025 and 2050, respectively. The cohort of Iranians, 0-14 years old, has been significantly declining since the 1990s. Based on various estimates, the proportion of the seniors will increase considerably in the next four decades. For instance, the number of seniors is estimated to reach 8.5 and 10.5 million by 2020 and 2025, respectively. The aging of the population will be somewhat more rapid in Iran than in other developing countries (Amini, Ingman & Sahaf, 2013).

Therefore, it is important to study the aging phenomenon on Iranian elderly and to understand it based on chronological age, taking into consideration the contexts, including cultural, historical, socio - economic and regional variables (Orimo et al.,2006).

A review of literature showed limited published study exploring cultural and socio-economic factors influencing changes in aging amongst women. Most reported studies have quantitatively investigated this topic. Additionally, most studies are confined to Western and European societies and have been carried out in social and cultural contexts that are very different from Eastern countries. Few quantitative studies have been undertaken in Iran and the qualitative aspects of this issue have not been studied to deeply understand the experiences of Iranian elderly women. Considering the fact that aging is a circuit process and is especially influenced by cultural and social contexts, and several confounding factors are involved in the interactions of the mentioned process, this study sought to provide a foundation for developing our knowledge about elderly women via the exploration of their experience.

## 2. Materials and Methods

This qualitative study was part of a more extensive study designed according to grounded theory method. Grounded theory is a method of qualitative research in the field of research and describes the phenomena experienced in natural environments such as hospitals, outpatient clinics and nursing homes. The purpose of this study was to find out whether the detail of practices, behaviors, beliefs and attitudes of individuals or groups is similar to their real life (Polit & Beck, 2006). A qualitative research aims to understand the social reality lies in the experiences of individuals, groups and cultures and seeks to discover the behavior, ideas, feelings and experiences of people and what are located at the center of their lives. This kind of research suggests that human’s experiences are dependent on the background of the experiences and cannot be detached from the time and situation (Corbin & Strauss, 2008). As a result, in this study we used a qualitative approach to explain the cultural and socio-economic factors which affect changes in elderly women.

Participants of the study were elder women living in private homes in Sari, Mazandaran province and Tehran, Tehran province in Iran. After obtaining informed written consent to participate in the study based on a purposeful, theoretical and snowball sampling technique, the eligible participants were recruited into the study (Polit & Beck, 2006).

Primary inclusion criteria were being female, aged 65 years and older, living in the home and no history of cognitive and mental disorders, cancer or physical illness that required hospitalization. The first sample was a 70-year-old woman with a high school diploma, widowed and housewife who lived alone in her private home in town. The next participant was a woman aged 67 years old introduced by the first participant and selected through snowball method based on the inclusion criteria. The remaining samples were recruited considering the inclusion criteria and were selected from elderly women with different characteristics in terms of age, educational level, occupation, marital status, residence location (city vs. rural areas) and socio-economic and cultural status. Elderly women living in the city and rural areas, older women who were married, single, widowed single, widowed while living with unmarried children, elderly widows living with another widow, or living independently at home with their children and widows with children living in their children’s home were enrolled. Following interview with the concepts generated by the interviews, it seemed interviews with people who came to live with the elderly could provide more information. Therefore, following gradual data gathering and analyzing information from research, for development of the data, the researcher did an interview with the daughter of an elderly woman who lived with her family at her mother’s home. Theoretical saturation of conceptual categories was accomplished after 20 interviews with 19 participants and the review of field notes taken by the researcher during the interviews.

Data collection instruments were semi-structured interviews and field notes. After each interview session, the recorded data was transcribed and field notes were rewritten by the researcher. In this study, information such as the participant’s appearance, non-verbal behaviors, interactions with other household members, roles, use of a cane, and the photos in home decorations all were recorded.

Several main questions that were repeated in almost all interviews were as follows: ‘Please describe your daily living activities from awaking in the morning to sleeping time at night’; ‘What are the issues and problems you are facing with?’; ‘If possible, can you explain more about your experiences of life in aging?’. The duration of interviews ranged from 38 to 110 minutes. Most of interviews ended at first session, and only in one case, the second interview was scheduled.

Data collection process, coding and analysis were performed concurrently and data analysis was performed using the recommended method by Corbin and Strauss (1998 and 2008).

In this method, three types of open coding, axial, and selective central was performed to discover the main categories, subcategories and core variable of the aging process. Open coding or substantive coding is conceptualizing on the first level of abstraction. Written data from field notes or transcripts were conceptualized line by line. The coding was often done in the margin of the field notes. The researchers goes back and forth while comparing data, constantly modifying, and sharpening the growing theory at the same time as she follows the build-up schedule of grounded theory’s different steps. By axial coding data were put back together in new ways after open coding, by making connections between categories. Selective coding was done after having found the core variable or what is thought to be the core, the tentative core. The core explains the behavior of the participants in resolving their main concern (Corbin & Strauss, 2008; Strauss & Corbin, 1998).

In this study, validation of data was established with prolonged engagement, member check, peer debriefing, triangulation, disconfirming evidence, and researcher credibility (Polit, Beck & Hungler, 2002). Moreover, elsewhere in the report as evidence of data dependency, the same words of the participants were used (Streubert & Carpenter, 2007). The researcher was trying to correctly interpret the findings with the idea of journalism (through the use of memos) and checking participates. In addition to leaving a footprint, utilized the new creative ideas to provide appropriate practical services in daily life of elderly people.

## 3. Results

Cultural and socio-economic factors were formed from 6 subcategories including previous cultural and socio-economic status, urban/rural life, companion status, beliefs and attitudes, higher responsibilities of women and women’s financial capability. According to the results, it seems that these factors were affecting all dimensions of physical, psychological, cultural and socio-economic of the elderly women. Other minor subcategories are shown in Table 1.

**Table 1:** Categories and subcategories of cultural and socio-economic factors on changes in aging among Iranian women

Cultural and socio-economic factors	
Previous cultural and socio-economic status	- Previous economic status
	- Previous companion status
	- Previous employment
Urban/rural life	Rural activity style
	Rural nutritional style
	More physical power of rural women
	More facilities in rural than past
Companion status	- Presences of companion
	- Type of companion
Beliefs and attitudes	- Beliefs about gender
	- Therapeutic and health care beliefs
Higher responsibilities of women	
Financial capability	- Healthcare costs
	- Children financial aids
	- A decrease in the cost of children’s education and marriage

### 3.1 Pervious Cultural and Socio-Economic Status

This subcategory emerged from minor subcategories including economic status, companion status and employment status. The data showed that the past lifestyle was as a predictive value as current lifestyle among the older women. About the effect of previous cultural and socio-economic status on current lifestyle, one participant said: ‘Well, my father was one of the great landlords in a good financial situation. I have the same inheritance income now. But it is not so good. However, I could handle my life by renting a house and so on. I have spent my life by traveling alone or with my family members and sometimes I go to entertainment events / parties’.

Also a retired participant expressed the importance of previous economic status: ‘… if I can find a job in my youthful days, I can take advantage of it during aging. Since I had a job during my youth, I do not need to ask my husband to give me some money to buy something every day. Now I am at old age. I have 6 grandchildren, one bride, two girls and their husbands. I have to buy birthdays gifts for all of them. Now, my husband and I pay our salaries one by one to provide the gifts. When we were young, we were away from each other. Now we are together and use each other’s incomes’.

Another participant also expressed about previous companion status: ‘It was just me and my husband. Our children lived separately in their own house. My husband was an employee of department of education. He was also poet and religious singer. We have always visited others at home and our house was a center for interaction with others. Come and go in our house is continued up to now. If only 5 people come to our house a day, we ask from ourselves: “what’s happened? Why others have left us alone”’.

Another elderly housewife woman expressed about previous employment: ‘If I was a man, I could work out, it was very good. Since I am a woman, I’m always at home and suffering from to be at home. Well, I’d like to have a job and worked out into the community. I’d like to work at home and outside too. People, who work out, could use their art and thoughts to do better work and could be successful in his/her life.’

Another elderly woman who was a retired nurse, said: ‘Well, my career really helped me know what I eat at this age. When I’m sick, I know what I should do. I know what is good for me or not. Using my knowledge, I could help myself and my friends. I do my daily work and I take my blood pressure pills by myself. I will seek the doctor if my attempts are failed. I provided all of my husband’s needs by myself. Having a job really causes someone to be mature. Working is really prefect.’

Another participant believed that: ‘Because I was a housewife from a younger age, I was not socialized, I did not know people. I taught all people around the world are perfect. Why should a person battle me when I am a widow woman who lives with her young children’.

### 3.2 Urban and Rural Life

This subcategory was formed from several minor subcategories including rural style activities, rural nutritional style, more physical power of rural women and more facilities in rural area compared to previous times. Most participants believed that changes in physical, mental and social status were influenced by their lifestyle. A participant said about the style of rural activities: ‘Because I have a garden, I personally do gardening and also participate with workers employed to do this job. After getting up in the morning, we prepare food for the workers. We also have livestock and farming. Summary, we have some cattle that make us busy. The day that we have workers on farm, I have to work hard. Activities in village are too much and so difficult, while the income are too low. We wake up early morning, say prayer and then go at work until sunset. We work, work…work even we feel pain, we still work. We can’t stop the work.’

The same participants said about the rural nutritional style: ‘But I eat too much rice. It is just my only mistake in regimen. The quantity of my diet is not less than before, because doing hard work in the village needs lots of energy and eating more consequently.’

Another 72 years old woman expressed about the more physical power in rural women: ‘I prepare all facilities for my guests alone. I prepare all of my needs by myself. I don’t need anyone helping me for my routine daily living. For example, when I invite my children in fasting month, I make food. Then they wash and dry the dishes; either my bride or my girls.’

This elderly lady, about the development of life in village compared to the past expressed: ‘At that time we did not have these facilities, there was many problems. Now we live easier than the past we did. I thank God. Already the work was very hurt and the life was very difficult. All family members lived with together. There were no facilities in the past. Our young people should not declare life isn’t easy today.’ She continued: ‘Feeling sad is now less prominent than in the past. Now everything is ready. When the guests arrive, we are happy. Everything is ready. The furniture, freezer and food are accessible’.

### 3.3 Companion Status

Companion status, is one of the other factors affecting the aging changes, was assessed in this study. This minor subcategory was formed by presence and type of companions. About necessity of companion, a participant said: ‘What does an elderly expect? An elderly needs to go outdoors, prefers to be with family members, to be with a partner and friends, not to leave alone, to be stable for living. If not, frequent thinking will lead to mental illness. Personally, if I don’t walk outside and stay home for a week, and have no meeting with others, I will be depressed’.

About the type of companions, an elderly woman stated: ‘In fact, I am very annoyed with someone who has stress. Someone who is suffering from stress and anxiety for anything, such as fear of crossing the road, or always suffering from conflict to do or not to do something, make me stressful. I try to have less contact with anxious people. Sometimes it is impossible to detach yourself from others and it bothers me. Conversely, I feel comfort with a number of people’.

Another elderly woman said: ‘I travel with my previous co-workers and classmates; I interact with people who have similar moral values’.

### 3.4 Beliefs and Attitudes

This subcategory includes codes of gender, attitudes, ideas and therapeutic and health care beliefs and attitudes. About the effect of gender on attitudes and beliefs a participant believed: ‘A man is free to select a partner, to go outdoors whenever he wants. Could I go outdoors at 12 midnight? I have to stay home. These differences cause emotional stress. Men are free. We are limited. Being free makes men to be health more than women. Certainly it affects physically and internal stress could be effective on everything’.

Another elderly woman said: ‘Women are more resilient than men. If a single woman survives to 85-90 years, she could live alone without thinking about getting married, but a man cannot do so. If I had died, my husband would have married. The men need partners to live. Well, women ignore mistakes too much, and can tolerate living alone’.

What we concluded from the data is that, having right and positive ideas can influence the application of proper strategies which lead to reduction of negative changes.

An elderly woman stated about the beliefs and attitudes of health care and therapeutics: ‘But we are always careless. Because we always have so much work to do, that causes that we ignore ourselves. Doctors are available, however we are careless to go to their office. A part of this ignorance is related to negligence and it is dangerous’.

Another participant said: ‘Since white bread causes obesity, I prefer to eat bran bread which does not cause obesity and is useful too. I often eat non-baked dinner. I have a glass of milk. Some foods like butter and jam are forbidden for us. Of course, I am not diabetic. I follow the diet because I don’t like to be fat’.

### 3.5 Higher responsibilities of Women

Employed elderly women who are retired now complained of this issue that they had many responsibilities in the past. They mentioned it as an etiological factor for aging-related injuries and diseases. One of the participants said about the great responsibilities of women: ‘We hope all men be healthy. Well, when a woman delivers a baby, lots of calcium and other elements transfers to the baby’s body and mother starts to become weak. Maybe just difficult baby deliveries cause low back pain in mother. About 90 percent of carriage, housework and shopping are done by women in households. Well, heavy burden of housekeeping is on the shoulders of women. So they have dubbed works and the hard work of household are not without adverse effects for the women’.

Another retired elderly said ‘Men do not take responsibility and do not have trouble! Certainly they are much better than us. Responsibility of a woman as a mother or housewife is very different. With someone like me who worked all day long; I had responsibility of home and kids. I had responsibility for supervising their educational classes and their lessons. I think men had no problems like we have. Their responsibility seems not to be much featured at home. Their job is just their responsibility and it is all they do’.

### 3.6 Financial Capability

In accordance to the findings observed, financial independence in the elderly not only provides them happiness, but also results in ability to change strategy to be effective in terms of their physical, mental-emotional, cultural and social-economic status. Financing power can be included dimensions of treatment costs, children financial support, and handling costs of children’s education and their marriage. Most participants were complaining of rising healthcare costs in this age. One of the elders said: ‘I told to my doctor that the cost of the tablets I take is too high for us at this age. I am a widow and have no extra income. We must live with employee salary. But within days we should pay for the pills which controls the pain and avoids bone deformities and builds bone materials. The doctor said “You could buy the Iranian pills, because each imported tablet is about 16,000 Rials”’. Another participant stated: ‘I always have a mobile pharmacy in my bag. I have no financial problems now. But I spend more for visiting the doctor, medicine and checkups than in the past’. About the financial support of children, an elderly expressed: ‘I have no income. My boys and girls are supporting me financially. They give financial aid to me monthly. They constructed the house I am living there now’.

Other participants stated about decreasing their children’s’ education and marriage expenses: ‘I’m satisfied with my life; I have enough income to spend. God bless my husband. I am benefited from what he prepared for me in the young age. I have already paid money for my children to get married. Now they are independent and there is no need for my support. Today, my life is better. I have no debit. I am living now’.

## 4. Discussion

In the present study, participants’ experiences showed that cultural and socio-economic status in the past, living in urban and rural area, companion status, beliefs and attitudes, more responsibilities of women and financial capacity were the cultural and socio-economic factors influencing changes in aging amongst Iranian women.

Categories organizing the cultural and socio-economic status of the elderly in the past in this study were: previous economic status, previous companion status and employment. In order to enrich the findings of this study, various participants to achieve a better view point of cultural and socio-economic conditions were enrolled.

Participants expressed being employed in the past in their life was a factor for success of various affairs of life, and believed that the current economic situation is the outcome of their previous social and economic status. It seems the theory of continuity confirms these findings. This theory expresses that the elderly continue the habits, adherence and values, especially in a way that they have chosen according to their social position to maintain sustainability. Therefore, knowing these factors, it can be predicted how they are old and what changes they experience (Eliopoulos, 2010; Mauk, 2006). Culture of an elderly person originates from social environment surrounding him/her and affects his/her attitude (Bryant et al.,2001). The aging process varies according to cultural change (Shin, Kim, & Kim, 2003). According to this analogy, all changes in elder life are caused by his/her culture. It is clear that separate assessments of various mental and biological aspects of life are meaningless. Each of these aspects is affected by another aspect, and this interaction is quite clear and obvious, especially in the elderly. So what we call mental and social life is only understood under human existential life situation. This situation and manner of life affects physical function. Because as before was mentioned, the analytical aspects of old age alone is not enough because each aspect had an impact on other aspects and other aspects are also affected. Therefore, study of the elderly should be comprehensive and complete. Aging can be understood only as a whole and is not only a biological fact but a true cultural, social and economic fact as well. Nevertheless, living conditions affect all aspects of life in the elderly (Bagheri-Nesami, 2012). Some similar studies have reported the effect of previous cultural and socio-economic status including culture, occupation, education and low income on physical disabilities (Chiu et al., 2005; Day, 2008; Jacelon, 2007), mental health (Grundy & Sloggett, 2003; Kawachi & Berkman, 2001), depression (Black et al.,2007), satisfaction of life (Nagalingam, 2007), health status, having a healthy old age (Bryant et al., 2001; Hinck, 2004; Matsubayashi et al., 2006; Shin et al., 2003; Strydom, 2005; van Manen, 2006) and successful aging (Matsubayashi et al.,2006).

In this study, the urban/rural life subclass was another factor influencing the cultural and socio- economic changes in the elderly. It included subcategories of rural activities, rural nutrition style, more physical power among rural women, and more access to more facilities in the village nowadays compared to the past. Rural participants in this study expressed that due to heavy work in the village, they have to do much more physical activity. Thus, compared to urban-living elderly, they need to consume more food in order to receive sufficient energy. Also, in comparison with the urban elderly, they believed that they are physically more powerful. Researches also show that the dominant culture of life in the village is hard work along with high activity (Dorfman et al., 2004; Smith, 2007). The rural elderly know working as a part of their life, even in the aging; especially work for agriculture, because it brings them the sense of well-being, physical activity and enjoyment. Desire for independence and unwillingness to change their previous lifestyle has also been dominant feature of rural culture. Thus, according to the continuum theory (Eliopoulos, 2010; Mauk, 2006) it can be said that rural-living people like to keep their previous lifestyle and remain active in the aging period. Other feature of rural culture is jointing the two categories of working and the families in rural areas (Dorfman et al., 2004; Goins, Williams, Carter, Spencer & Solovieva, 2006). The elderly in rural areas compared with urban elderly also benefit from more resources and social support, and this issue will contribute more to their mental health. This study showed that self-reporting about health status is similar between elderly in both urban and rural areas and the amount of high quality and useful food intake strategy, there was no differences between the two elderly groups. Another similar result with the present study was accessibility to facilities like freezer that prepare opportunities for rural elderly to choose and having the necessary foods. It seems rural elderly are overcome on their nutritional requirement (Smith, 2007). It is notable that this study reported a difference of more than five years of life for elderly in rural areas compared to urban elderly.

In the present study, participants expressed the companion status as another factor influencing cultural and social-economic aspects. The participants believed that communication is a source of support by others and improve physical, mental and social status. According to experiences of key informants in this study, elderly chose their communicators based on their mental and psychological characteristics. So, most participants preferred to cut their relationship with someone who is anxious. Social capital has positive and negative effects on the elderly. If the resources or the people are appropriate, the elderly will be successful; otherwise the individual may be suffering from loneliness or social separation (Cannuscio, Block & Kawachi, 2003). The findings of other studies also indicate the fact that communication with people benefit mental and physical health (Gilhooly et al., 2007; Kawachi & Berkman, 2001; Kunzmann, Little, & Smith, 2000; Walker & Hiller, 2007), prevent loss of cognitive impairment such as Alzheimer’s and dementia (Fratiglioni, Paillard-Borg & Winblad, 2004; Walker & Hiller, 2007), increase perfect sense, being independent and productive (Cannuscio et al.,2003) and increase longevity (Giles, Glonek, Luszcz & Andrews, 2005; Walker & Hiller, 2007) in the elderly.

Other contributing factors influencing changes in aging amongst Iranian elderly women were beliefs and attitudes. The results showed the shape of health behaviors, medical follow-ups, adherence to health advice, the prevention of changes in aging and enjoyment of health beliefs amongst Iranian elderly women. It is believed that attitudes, subjective norms and perceived ability to control the behavior of the elderly are mixed together to create a goal to perform a behavior. Therefore, beliefs and attitudes, expectations and behavior formed and influenced by the changes in aging can affect adopted behavior (Ramamurti, 1997; Reichstadt et al., 2007; Sarkisian et al.,2002). Also, the attitude among the elderly is a predictive important factor for the understanding of health, feeling close to death and problems of end of life was reported (Torsch & Ma, 2000). The data analysis related to a study showed that there was no significantly a difference between successful and unsuccessful elderly as viewpoints of numbers rate of life events experienced. But unsuccessful older people were more negative in their experience and feel the experience has had a negative impact on their lives. Based on the results of this study, attitudes and beliefs of the elderly are the main issues affecting changes in the period of life (Reichstadt et al.,2007).

Another category in the present study, as factors contributing to the cultural and socio-economic changes of aging, was the more responsibilities of women than men. Two groups of participants had complained about responsibilities more than others. The two groups were; rural elderly women and women who work outdoors. The contributors stated working together outdoors and inside the home caused the physical and psychological erosion among them. Feminist theorists believed that most of the activities women do, influenced by the culture and norms of society. Today, with the rise of modernity, the lives of women must be changed, while today women are under past man-dominated culture and therefore they have not equal rights. Theorists believe that what these elderly women do, it is not what others expect of her, or should do, and this is a reason of there are being under pressure, suffering and hardship and increase in women’s responsibility. According to the feminist theory in comparison with men’s duties, taking care of families with having a mother relationship with children is a moral concept like justice and dignity that is far off human rights law (Utz & Nordmeyer, 2007). It seems women have a sense of responsibility to their families and children. Women’s work is a kind of sacrifice and opposite of the perspectives of the feminist theory; this is not a default from woman who involves her in trouble. Although it is difficult, it is possible by family support and government protection. Current laws to help women, such as labor rights, breast feeding, retirement with 25 years’ service, etc. in Iran expresses itself in honoring the women.

Participants’ experiences showed that financial power is one of the categories influencing cultural and socio-economic changes in the elderly. Elderly women expressed factors such as medical expenses, financial aids of children, decreasing children’s’ education and married expenses’ costs as important factors affecting financial power. Because through supporting roles for family and children, older women while enjoying financial assistance to the relatives and next-generation, will be able to achieve capacity, respect and dignity of old age (Fiksenbaum, Greenglass & Eaton, 2006; Nagalingam, 2007; Ravanipour, Salehi, Taleghani, Abedi & Schuurmans, 2008; Strydom, 2005). Also, according to the participants’ experiences and findings of other studies, older women, when faced with financial problems, may experience the consequences of social separation, depression and fear of future (Fiksenbaum et al., 2006; Ravanipour et al., 2008; Strydom, 2005). So, based on what mentioned above, changes in aging are affected by cultural and socio-economic contexts of life.

The results of our study can help health care providers to be attuned to the needs of Iranian elderly women, delivering them more suitable health care services.
